# The Relationship Between Weight Indices and Blood Levels of Immunosuppressive Drugs in Renal Transplant Recipients

**DOI:** 10.5812/ijpr-146619

**Published:** 2024-09-16

**Authors:** Shirinsadat Badri, Bozorgmehr Dadkhah-Tehrani, Abdolamir Atapour, Shahrzad Shahidi, Mojgan Mortazavi, Tahereh Gholipourshahraki

**Affiliations:** 1 Department of Clinical Pharmacy and Pharmacy Practice, Isfahan University of Medical Sciences, Isfahan, Iran; 2 Isfahan Kidney Diseases Research Center, Isfahan University of Medical Sciences, Isfahan, Iran; 3 Pharmacy Students’ Research Committee, Isfahan University of Medical Sciences, Isfahan, Iran

**Keywords:** Calcineurin Inhibitors, MTOR Inhibitors, Drug Monitoring, Kidney Transplantation, Body Weight

## Abstract

**Background:**

Calcineurin inhibitors and mammalian target of rapamycin (mTOR) inhibitors are essential for maintaining transplanted organs. However, determining the appropriate dosage and predicting blood concentrations of these drugs based solely on net body weight may be inadequate. Previous studies have presented contradictory results regarding the impact of obesity on drug concentrations and transplant success.

**Objectives:**

This study aims to evaluate various weight indices to identify the most reliable indicator of weight that correlates with the blood levels of drugs used in organ transplantation.

**Methods:**

This retrospective descriptive study included patients from nephrology clinics affiliated with Isfahan University of Medical Sciences who were taking calcineurin and/or mTOR inhibitor drugs. Data extracted from medical records included demographic and clinical information, such as height, weight, and various weight indices (total/ideal/adjusted body weight, lean body mass (LBM), Body Mass Index, and predicted normal weight), as well as blood levels of immunosuppressive drugs at each patient's visit. The dosages of each drug (mg/kg) were analyzed to determine which weight indices best correlated with the obtained blood concentrations, using the Generalized Estimating Equation (GEE) model with logistic regression, an independent correlation matrix, and a binary distribution for data analysis.

**Results:**

The study analyzed the medical records of 71 patients. Trough (C0) concentrations of drugs were evaluated in relation to each weight index, and odds ratios (OR) were calculated for statistical comparison. All weight indices increased the likelihood of achieving appropriate concentrations for cyclosporine, tacrolimus, and sirolimus. Drug dosing based on LBM (OR: 1.028), ideal body weight (OR: 1.075), and total body weight (OR: 1.041) showed the strongest correlations with achieving proper blood levels for cyclosporine, tacrolimus, and sirolimus, respectively.

**Conclusions:**

Integrating various weight indices for calculating individualized doses (mg/kg) of each immunosuppressive drug increases the likelihood of achieving appropriate blood concentrations. However, the optimal weight index varies for each drug. Further studies, particularly those incorporating therapeutic drug monitoring (TDM) plans in transplant centers, are warranted to validate and generalize these findings, providing a potential avenue for improving immunosuppressive therapy and enhancing transplant outcomes.

## 1. Background

Renal transplantation is a life-saving strategy for patients suffering from end-stage renal disease (ESRD) (1). The success of a renal transplant plays a critical role in restoring kidney function, improving the quality of life, prolonging graft survival, reducing complications associated with dialysis, enhancing survival rates, and providing a cost-effective long-term treatment option (2, 3). Immunosuppressive drugs are essential for ensuring the success of a renal transplant. By suppressing the immune response, these drugs reduce the risk of organ rejection and promote long-term graft survival (4-6). Immunosuppressive regimens typically consist of a combination of drugs, including calcineurin inhibitors (such as cyclosporine or tacrolimus), antimetabolites (such as mycophenolate mofetil or azathioprine), mammalian target of rapamycin (mTOR) inhibitors (such as sirolimus), and corticosteroids. While these drugs effectively suppress the immune system, they can also have various effects on the body, including metabolic alterations (7, 8).

In addition to the challenges of renal transplantation, obesity presents special considerations that require attention (9). Obesity has become a significant health concern worldwide, with its prevalence steadily increasing over the past decades (10, 11). In the context of kidney transplantation, obesity poses unique challenges that warrant careful consideration. Obesity is associated with an increased risk of developing ESRD, the primary indication for kidney transplantation. Obese individuals are more likely to develop conditions such as diabetes, hypertension, and chronic kidney disease, which can ultimately progress to ESRD (11, 12). As a result, the prevalence of obesity among renal transplant candidates is rising, highlighting the need to understand its impact on transplantation outcomes and immunosuppressive therapy (13).

It is well-established that weight indices, such as Body Mass Index (BMI), body weight, and body composition, can significantly impact drug pharmacokinetics and pharmacodynamics (14). Adipose tissue, a major component of body weight, has metabolic activity and can act as a reservoir for lipophilic drugs, altering their distribution and leading to changes in drug concentrations in the blood. Furthermore, obesity is often associated with alterations in drug-metabolizing enzymes and transporters, which can affect drug metabolism and elimination (15, 16). Achieving optimal immunosuppression is crucial in renal transplantation to prevent rejection of the transplanted organ. Immunosuppressive drugs are vital components of post-transplantation care, and their dosages need to be carefully adjusted to maintain a delicate balance between preventing rejection and minimizing drug-related adverse effects. Understanding the relationship between weight indices and blood levels of immunosuppressive drugs is essential for tailoring individualized treatment regimens and optimizing immunosuppression in renal transplant patients (17, 18).

## 2. Objectives

In this study, we aim to investigate the relationship between weight indices and blood levels of immunosuppressive drugs in renal transplant patients. By exploring this relationship, we hope to contribute to the existing body of knowledge and provide valuable insights for optimizing immunosuppressive therapy in this patient population.

## 3. Methods

### 3.1. Study Design

This research was a retrospective observational study designed to explore the intricate relationship between various weight indices and the blood levels of immunosuppressive drugs in renal transplant patients. The study aimed to elucidate the impact of different weight indices on the concentrations of these critical drugs, with the goal of optimizing therapeutic drug monitoring strategies and improving treatment outcomes. The data used in this investigation were obtained from three private nephrology clinics specializing in the care of primary kidney transplant recipients. The data collection period spanned one year, from September 2019 to September 2020, to encompass a substantial sample size and capture longitudinal variations in drug concentrations.

### 3.2. Participants

The selection of participants for this study followed stringent inclusion and exclusion criteria to ensure the relevance and integrity of the research findings. Renal transplant patients meeting the following inclusion criteria were considered eligible for the study: (1) age 18 years or older; (2) having undergone a primary kidney transplant with at least six months elapsed since the transplantation procedure; (3) demonstrating a serum creatinine concentration below 2 mg/dL; (4) displaying serum creatinine changes within the past month of less than 30%; (5) receiving treatment with either calcineurin inhibitors (such as cyclosporine or tacrolimus) or sirolimus; and (6) attending a minimum of three outpatient visits for follow-up care.

Conversely, individuals with a history of acute kidney injury, as defined by the kidney disease: Improving global outcomes (KDIGO) criteria (8), during the month prior to the study initiation were excluded from the study population. Additionally, patients with a history of treatment for acute graft rejection or infection in the month leading up to the commencement of the study were also excluded.

### 3.3. Data Collection

For comprehensive data collection, baseline demographic and clinical characteristics were meticulously recorded for all study participants. The essential demographic information collected included age, gender, and weight indices. Additionally, detailed information regarding the immunosuppressive drug regimen was gathered for each participant. This included data on the dosage, brand, or formulation of the prescribed calcineurin inhibitors (e.g., cyclosporine, tacrolimus) and sirolimus.

Specific weight measurements were used to assess the relationship between weight indices and drug concentrations. The optimal target therapeutic levels for trough concentration (C0) were defined as follows: Cyclosporine 75 - 150 ng/mL, tacrolimus 5 - 10 ng/mL, and sirolimus 5 - 15 ng/mL (19, 20). The weight indices considered in this study included total body weight (TBW), BMI, ideal body weight (IBW), adjusted body weight (AjBW), lean body mass (LBM), and predicted normal weight (PNWT). To calculate these indices, precise formulas based on weight, height, and gender were applied, ensuring accurate and standardized measurements across the study cohort. Table 1 presents the equations used to determine the weight indices in the study (21).

**Table 1.** Equations for Weight Indices Calculation

Weight Indices	Equation
**TBW**	Patient's actual weight measured in kilograms (kg)
**BMI**	TBW/Ht^2^ (kg/m^2^)
**IBW**	
Male	50 + [0.9 × (Ht (cm) - 154)]
Female	45.5 + [0.9 × (Ht (cm) - 154)]
**LBM**	
Male	[0.407 × TBW + 0.267 × Ht (cm)] - 19.2
Females	[0.252 × TBW + 0.473 × Ht (cm)] - 48.3
**AjBW**	IBW + 0.4 [(TBW - IBW)]
**PNWT**	
Male	[(1.57 × TBW) - (0.0183 × BMI × TBW)] - 10.5
Female	[(1.75 × TBW) - (0.0242 × BMI × TBW)] - 12.6

Abbreviations: TBW, total body weight; BMI, Body Mass Index; IBW, ideal body weight; LBM, lean body mass; AjBW, adjusted body weight; PNWT, predicted normal weight; Ht, height; kg, kilogram; m, meter; m^2^, square meter; cm, centimeters.

### 3.4. Statistical Analysis

In this study, the data obtained from the participants were subjected to thorough statistical analysis to explore the relationship between weight indices and blood levels of immunosuppressive drugs. Descriptive statistics were employed to summarize the demographic, clinical, and laboratory characteristics of the study cohort. These characteristics included various variables, such as weight indices, drug dosages, specific drug brands, trough concentration (C0) levels of immunosuppressive drugs, and serum creatinine concentrations.

The statistical software statistical package for the social sciences (SPSS) version 16.0 (developed by IBM in Chicago, Illinois, United States) was used for the data analysis. To explore the association between weight indices and the likelihood of achieving appropriate drug concentrations, the generalized estimating equations (GEE) model was employed. This statistical model is particularly suitable for analyzing data from repeated measures, which is common in longitudinal studies or when multiple measurements are taken from the same individual over time. The GEE model effectively accounted for repeated measures over time, ensuring robust and valid results.

The GEE model utilized a logit link function and an independent correlation matrix, with a binary distribution applied to examine the relationship between weight indices and drug concentrations. The odds ratio (OR) of achieving appropriate drug concentrations was calculated for each weight index. An odds ratio greater than 1 indicated a higher chance of attaining appropriate drug concentrations, whereas an odds ratio less than 1 suggested a lower likelihood.

Statistical significance was determined using the P-value, with a threshold set at P < 0.05.

### 3.5. Ethical Considerations

This study was conducted following the approval of the research ethics committee of Isfahan University of Medical Sciences (ethics code: IR.MUI.MED.REC.1398.213). Before participation, all subjects were fully informed about the study's objectives and methods, and written informed consent was obtained from each participant. To ensure confidentiality, all recorded information from patients' files was kept under the strict supervision and responsibility of the study administrators.

## 4. Results

A total of 71 renal transplant patients were included in the study, with a mean age of 52.66 years. The majority of participants were male (70.42%). Based on BMI classifications, the distribution of the patients was as follows: 7 patients (9.86%) were underweight (BMI < 18.5), 36 patients (50.70%) had normal weight (BMI 18.5 - 24.9), 8 patients (11.26%) were overweight (BMI 25 - 29.9), and 20 patients (28.17%) were obese (BMI ≥ 30). The baseline characteristics of the participants are summarized in Table 2. 

**Table 2.** Baseline Characteristics of Study Participants

Characteristics	Mean ± SD (Range)	
**Age (y)**	52.66 ± 11.78 (27 - 85)	
**Weight (kg)**	68.11 ± 18.91 (33 - 91)	
**Height (m)**	1.68 ± 0.11 (1.41 - 1.87)	
**Drug Name**	**Daily Dose (mg)**	**Serum Concentration (ng/mL)**
**Cyclosporine (n = 53)**	221 ± 88.53 (50 - 450)	148 ± 75.46 (35 - 556)
**Tacrolimus (n = 19)**	3.77 ± 0.9 (1.5 - 5)	9 ± 2.30 (3.4 - 23)
**Sirolimus (n = 16)**	1.79 ± 0.79 (1 - 3)	8.75 ± 2.44 (4.1 - 11.5)

Abbreviations: BMI, Body Mass Index; kg, kilogram; m, meter; m^2^, square meter; SD, standard deviation.

In Table 3, the distribution of patients based on drug concentration levels for cyclosporine, tacrolimus, and sirolimus is presented. The table shows the percentage of patients with therapeutic, under-therapeutic, and over-therapeutic levels for each drug.

**Table 3.** Distribution of Patients Based on Drug Concentration Levels

Drug Name	Therapeutic Level (ng/mL)	Patients with Therapeutic Levels	Patients with Under Therapeutic Levels	Patients with Over Therapeutic Levels
**Cyclosporine**	75 - 150	16%	62%	22%
**Tacrolimus**	5 - 10	1%	35%	64%
**Sirolimus**	5 - 15	41%	36%	23%

Table 4 presents the average anthropometric indicators, along with the average values at appropriate and inappropriate drug concentration levels for each drug separately. Statistical significance was assessed using the P-value, with values lower than 0.05 indicating significant differences in the means.

**Table 4.** Average Anthropometric Indicators and Drug Concentration Levels

Drug Name	Weight Indices					
	TBW	BMI	IBW	LBM	AjBW	PNWT
**Cyclosporine**	64.81 ± 11.90 (33 - 91)	22.64 ± 2.83 (13.73 - 35.24)	64.44 ± 9.87 (39.75 - 80.7)	52.21 ± 8.23 (30.5 - 63.12)	64.6 ± 10.02 (44.53 - 73.34)	63.97 ± 11.73 (33.02 - 79.51)
Mean (approp) ^a^	63.51	22.48	63.43	51.26	63.46	62.62
Mean (inapprop) ^b^	66.92	22.90	66.08	53.75	66.41	66.17
P-value	< 0.001	0.012	< 0.001	< 0.001	< 0.001	< 0.001
**Tacrolimus**	59.83 ± 10.87 (37 - 77)	24.10 ± 4.27 (16.01 - 63.65)	54.47 ± 6.13 (42.42 - 67.34)	46.86 ± 6.41 (33.11 - 58.60)	56.61 ± 6.78 (44.53 - 70.80)	56.34 ± 9.14 (36.75 - 73.09)
Mean (approp)	58.69	25.03	50.87	44.99	54.00	53.69
Mean (inapprop)	60.40	23.67	56.21	47.77	57.88	57.64
P-value	0.623	0.387	0.002	0.149	0.045	0.148
**Sirolimus**	56.53 ± 8.85 (46 - 80)	23.82 ± 5.98 (16.76 - 36.60)	66.61 ± 10.90 (42.42 - 84.90)	52.24 ± 9.89 (39.32 - 62.75)	65.20 ± 7.83 (48.52 - 74.54)	62.98 ± 12.59 (46.60 - 78.45)
Mean (approp)	53.89	23.45	61.69	49.25	61.04	56.03
Mean (inapprop)	58.06	24.11	70.46	54.58	68.46	64.06
P-value	0.144	0.722	0.013	0.088	0.032	0.08

Abbreviations: SD, standard deviation; TBW, total body weight; BMI, Body Mass Index; IBW, ideal body weight; LBM, lean body mass; AjBW, adjusted body weight; PNWT, predicted normal weight.

^a^ Concentration within the therapeutic target level.

^b^ Concentration out of the therapeutic target level.

For cyclosporine, significant differences were observed in all weight indices between patients with appropriate drug concentrations and those with inappropriate concentrations. Patients with appropriate concentrations had lower average weight indices. In contrast, for tacrolimus, only the IBW showed a statistically significant difference between patients with appropriate and inappropriate drug concentrations (50.87 kg vs. 56.21 kg, P = 0.002). Other weight indices did not exhibit significant differences between the two groups.

Similarly, for sirolimus, significant differences were observed in IBW (61.69 kg vs. 70.46 kg, P = 0.013) and AjBW (49.25 kg vs. 54.58 kg, P = 0.088) between patients with appropriate and inappropriate drug concentrations. However, other weight indices did not show statistically significant differences between the two groups.

The GEE model was used to analyze the relationship between weight indices and drug concentrations. This study examined the odds ratio of achieving appropriate versus inappropriate drug concentrations for each weight indicator in the study population. The results indicated that using each weight index could effectively increase the chance of attaining appropriate drug concentrations, with variations observed for different drugs, as depicted in the following tables.

As shown in Table 5, except for TBW, all other weight indicators increased the odds of achieving appropriate drug concentrations for cyclosporine, with LBM exhibiting the best performance. Similarly, for tacrolimus, all the weight indicators showed a significant increase in the odds of achieving appropriate drug concentrations, with IBW demonstrating the highest efficacy. For sirolimus, all the weight indicators significantly increased the odds of proper drug concentrations, with TBW yielding the best results. Notably, due to the separation of results for each medication, the P-values for these differences across the three drugs did not reach the level of statistical significance.

**Table 5.** Analytical Table of Results for Cyclosporine, Tacrolimus, and Sirolimus

Drug Name	Weight Indices					
	TBW	BMI	IBW	LBM	AjBW	PNWT
**Cyclosporine**						
Odds ratio	.013	1.009	1.027	1.028	1.023	1.020
Confidence interval	0.987-1.040	0.929-1.096	0.992-1.063	0.989-1.069	0.990-1057	0.992-1.048
P-value	0.317	0.836	0.128	0.164	0.168	0.163
**Tacrolimus**						
Odds ratio	1.003	1.001	1.075	1.027	1.012	1.019
Confidence interval	0.992-1.013	0.976-1.025	0.989-1.169	0.979-1.079	0.986-1.038	0.985-1.054
P-value	0.631	0.958	0.089	0.276	0.362	0.275
**Sirolimus**						
Odds Ratio	1.041	1.001	1.028	1.023	1.024	1.018
Confidence interval	0.942 - 1.152	0.850 - 1.179	0.944 - 1.120	0.922 - 1.134	0.936 - 1.120	0.941 - 1.102
P-value	0.429	0.992	0.523	0.667	0.609	0.652

Abbreviations: TBW, total body weight; BMI, Body Mass Index; IBW, ideal body weight; LBM, lean body mass; AjBW, adjusted body weight; PNWT, predicted normal weight.

## 5. Discussion

This study investigated the relationship between different weight indices and plasma concentrations of immunosuppressive drugs in renal transplant patients. Our analysis revealed distinct patterns of association between weight indices and drug concentrations for different immunosuppressive agents. The GEE model showed that all weight indices increased the likelihood of achieving appropriate drug concentrations for cyclosporine, tacrolimus, and sirolimus, with lean body weight (LBW), IBW, and TBW demonstrating the best performance for each drug, respectively.

The increasing number of medical and paramedical specialties has led to more health professionals participating in the clinical care of specific patients, particularly transplant recipients. Recent studies have emphasized the constructive role of pharmacists as members of the treatment team in hospital transplant departments (22-24).

In the last decade, the number of immunosuppressive drugs and other medications used in transplantation has increased significantly, leading to more complex drug regimens, potential interactions, complications, and higher costs (25). Several studies have reported the association of obesity with a wide range of post-transplant complications, including reduced graft survival, kidney complications, delayed organ function, and reduced patient survival (9, 25).

The study by Singh et al. shows that obesity has an insignificant effect on post-transplant results. This study found that obesity was not associated with major short- and long-term post-transplantation complications, aside from minor post-transplantation complications and an increased hospital stay. The results of this retrospective study indicated that obesity primarily increases complications related to surgery and the duration of hospitalization. Although there was a trend toward delayed organ function, acute kidney injury, and increased serum creatinine in obese subjects, these differences were not statistically significant (26).

It has been suggested that obesity can affect the achievement of optimal therapeutic concentrations of immunosuppressive drugs. In a retrospective study, Hortal et al. examined the relationship between obesity and cyclosporine concentration in 28 patients, 14 of whom were obese. They measured C0 and C2 concentrations and found that C0 was similar in both obese and non-obese groups. This study highlights the significant impact of patient weight on cyclosporine bioavailability, half-life, and clearance, suggesting that a patient's IBW should primarily be used to adjust cyclosporine dosage. The study provides a comprehensive understanding of how biophysical factors, such as patient weight, are critical in transplant scenarios, particularly regarding treatment measures like cyclosporine dosage (27).

Another study aimed to investigate the impact of obesity and overweight on cyclosporine blood levels in patients. A total of 27 patients were included in the survey, with 778 visits being evaluated. The patients were categorized into different groups based on their BMI percentiles. Clinical and laboratory parameters, including serum creatinine levels, glomerular filtration rate (GFR), and proteinuria, were measured and compared between the groups. The findings of this study suggest that weight gain, particularly obesity and overweight, is associated with poorer renal function but not necessarily with more significant proteinuria. Additionally, smaller cyclosporine doses were found to be adequate in maintaining blood levels comparable to those in lean patients. The elevated serum creatinine levels and reduced GFR during periods of obesity and/or overweight suggest impaired renal function in these individuals. This may be attributed to the underlying mechanisms of obesity, such as inflammation and oxidative stress, which can negatively affect renal function (28).

Similarly, researchers evaluated the influence of body weight on the pharmacokinetics of cyclosporine in adult uremic candidates for renal transplantation (29). A total of 45 patients underwent detailed nutritional assessment and pharmacokinetic analysis. When normalized by IBW, body surface area, or as absolute values, pharmacokinetic analyses revealed no significant differences in the bioavailability, elimination half-life, clearance, or apparent steady-state volume of distribution of cyclosporine between obese and non-obese patients. However, when dosed according to TBW, obese recipients had higher mean serum cyclosporine trough levels compared to non-obese recipients on day seven after transplantation. Therefore, to achieve comparable drug concentrations during the early transplant period, cyclosporine dosing should be based on IBW for obese patients.

Han et al. investigated the relationship between tacrolimus concentrations and body composition markers in kidney recipients. The baseline characteristics of the 18 patients recruited from Seoul National University Hospital were described. The study found differences in tacrolimus concentrations between the high and low-fat mass groups at 0 and 4 hours. Additionally, lean mass analysis revealed differences in tacrolimus concentrations. These findings indicate a potential association between body composition markers and tacrolimus concentrations in kidney recipients, which may have implications for optimizing tacrolimus dosing in this population. However, further research and intervention studies are needed to confirm the significance of these correlations (30).

Researchers used routine monitoring results to develop a predictive model for the area under the concentration versus time curve (AUC) of cyclosporine in renal transplant patients (31). They concluded that obesity affects the pharmacokinetics of cyclosporine after kidney transplantation; therefore, dose adjustment in obese patients should not be based on a linear relationship between daily dose and AUC versus time.

A study on pediatric renal transplant patients investigated the pharmacokinetics of cyclosporine and found that body weight was one of the factors influencing the apparent central volume of distribution of cyclosporine. This suggests that weight indices may impact the blood levels of cyclosporine in these patients (32).

Contrary to previous studies, a study aimed to investigate the outcomes of renal transplantation in obese recipients compared to non-obese recipients. The study population included 127 obese patients (BMI > 30 kg/m²) and a matched non-obese control group of 127 recipients. The follow-up period was 58.9 ± 40 months. Non-obese patients had significantly greater survival rates (89% vs. 67% in obese patients) at five years and experienced fewer deaths during the follow-up period. Cardiac disease was the leading cause of death in the obese group. There were no significant differences between the groups in terms of graft function or rejection rates. However, obese patients had more complications per patient and a higher incidence of post-transplant diabetes. Despite receiving less cyclosporine, obese recipients showed similar blood levels. The study concludes that obesity primarily impacts patient mortality due to cardiac events, and careful pretransplant screening for ischemic heart disease is essential for high-risk obese patients. Weight reduction before transplantation is recommended for all patients, especially those with a history of cardiac disease (33).

A review by Jindal and Zawada mentioned that obesity is a significant health problem in both the Western world and developing countries today. This review shows that obesity is related to delayed organ function, though the exact cause is unclear. There is wide disagreement between centers about the long-term outcomes of obese patients after successful kidney transplantation. It is also suggested that a multi-faceted approach is needed to reduce obesity before and after kidney transplantation. Obesity has been associated with increased C0 concentrations and nephrotoxicity in immunosuppressive regimens based on cyclosporine, which can be less than in regimens based on tacrolimus and sirolimus (34).

The study by Dashti-Khavidaki et al. assessed tacrolimus dosing in Iranian kidney transplant patients within the first three weeks post-transplant. Their findings underscore the necessity of individualized tacrolimus dosing in this population. The results showed that patients required lower daily doses than recommended to reach target blood levels, with females needing higher doses than males to achieve similar levels (35).

In a recently published review paper, the authors discuss the survival benefit of kidney transplantation compared to remaining on the waitlist for obese patients. Data from the United States Renal Data System (USRDS) between 1995 and 2007 showed that transplant recipients experienced improved long-term survival and quality of life compared to those who remained on dialysis. The extent of the survival benefit varied based on the patient's BMI. Overall, the paper highlights the importance of considering obesity in the context of kidney transplantation and emphasizes the potential benefits of transplantation for obese individuals (9).

The results of this study indicate that BMI emerges as a more stable predictor for appropriate drug dosing compared to other weight indices. This suggests that using BMI to determine drug dosage may lead to more consistent drug concentrations and improved therapeutic outcomes. The GEE analysis further demonstrates that each weight index can increase the likelihood of achieving appropriate drug concentrations for cyclosporine, tacrolimus, and sirolimus, with varying degrees of efficacy for each drug.

The study's insights have practical implications for clinical practice, highlighting the importance of considering specific weight indices when prescribing immunosuppressive drugs for renal transplant patients. Tailoring drug dosages based on individual weight characteristics can help minimize the risk of drug-related adverse effects while maintaining adequate immunosuppression.

Despite the valuable contributions of this research, it is essential to acknowledge the study's limitations. The relatively small sample size may limit the generalizability of the findings, warranting further investigation with larger cohorts to validate and expand on these results. Additionally, the study was conducted in specific clinical settings, and variations in patient populations and drug regimens may influence the relationship between weight indices and drug concentrations in different contexts. Another possible limitation of this study is that it does not assess the potential association between weight indices and clinical outcomes, including acute allograft rejection.

In conclusion, this study sheds light on the crucial relationship between weight indices and blood levels of immunosuppressive drugs in renal transplant patients. The findings emphasize the importance of individualized drug dosing based on specific weight indices to optimize immunosuppressive therapy and enhance transplant outcomes. While certain weight indices may exhibit stronger associations with drug concentrations for specific medications, a comprehensive assessment of all relevant indices is essential for personalized dosing and improved therapeutic outcomes. More extensive multicenter studies with diverse patient populations are encouraged to strengthen the evidence base and establish standardized guidelines for individualized drug dosing in renal transplant recipients. Collaborative efforts between healthcare professionals, including pharmacists, nephrologists, and transplant surgeons, can facilitate the implementation of tailored therapeutic approaches to improve long-term transplant outcomes and patient well-being.

## Data Availability

The dataset presented in the study is available on request from the corresponding author during submission or after publication.

## References

[1] Camilleri B, Pararajasingam R, Buttigieg J, Halawa A (2020). Renal transplantation in the elderly: Outcomes and recommendations.. Transplant Rev (Orlando)..

[2] Gaston RS (2016). Improving Long-Term Outcomes in Kidney Transplantation: Towards a New Paradigm of Post-Transplant Care in the United States.. Trans Am Clin Climatol Assoc..

[3] Garcia GG, Harden P, Chapman J, The World Kidney Day Steering Committee (2012). The Global role of kidney transplantation.. J Nephropathol..

[4] Muntean A, Lucan M (2013). Immunosuppression in kidney transplantation.. Clujul Med..

[5] Roberts MB, Fishman JA (2021). Immunosuppressive Agents and Infectious Risk in Transplantation: Managing the "Net State of Immunosuppression".. Clin Infect Dis..

[6] Namazi S, Sagheb MM, Karimzadeh I (2012). Adverse reactions of immunosuppressive drugs in Iranian adult kidney transplant recipients.. Exp Clin Transplant..

[7] Hartono C, Muthukumar T, Suthanthiran M (2013). Immunosuppressive drug therapy.. Cold Spring Harb Perspect Med..

[8] Chadban SJ, Ahn C, Axelrod DA, Foster BJ, Kasiske BL, Kher V (2020). KDIGO Clinical Practice Guideline on the Evaluation and Management of Candidates for Kidney Transplantation.. Transplantation..

[9] Chang JH, Mushailov V, Mohan S (2023). Obesity and kidney transplantation.. Curr Opin Organ Transplant..

[10] Diwan TS, Cuffy MC, Linares-Cervantes I, Govil A (2020). Impact of obesity on dialysis and transplant and its management.. Semin Dial..

[11] Friedman AN (2022). Obesity in CKD: A Promising Path Forward.. Clin J Am Soc Nephrol..

[12] Chen Y, Dabbas W, Gangemi A, Benedetti E, Lash J, Finn PW (2021). Obesity Management and Chronic Kidney Disease.. Semin Nephrol..

[13] Oniscu GC, Abramowicz D, Bolignano D, Gandolfini I, Hellemans R, Maggiore U (2021). Management of obesity in kidney transplant candidates and recipients: A clinical practice guideline by the DESCARTES Working Group of ERA.. Nephrol Dial Transplant..

[14] Hanley MJ, Abernethy DR, Greenblatt DJ (2010). Effect of obesity on the pharmacokinetics of drugs in humans.. Clin Pharmacokinet..

[15] Tan A, Wilson S, Sumithran P (2022). The application of body mass index-based eligibility criteria may represent an unjustified barrier to renal transplantation in people with obesity.. Clin Obes..

[16] Foucher Y, Lorent M, Albano L, Roux S, Pernin V, Le Quintrec M (2021). Renal transplantation outcomes in obese patients: a French cohort-based study.. BMC Nephrol..

[17] Srinivas TR, Meier-Kriesche HU (2008). Minimizing immunosuppression, an alternative approach to reducing side effects: objectives and interim result.. Clin J Am Soc Nephrol..

[18] Cheung CY, Tang SCW (2022). Personalized immunosuppression after kidney transplantation.. Nephrol (Carlton)..

[19] Kidney Disease: Improving Global Outcomes Transplant Work G (2009). KDIGO clinical practice guideline for the care of kidney transplant recipients.. Am J Transplant..

[20] Zeind CS, Carvalho MG, Cheng JW, Zaiken K, LaPointe T (2023). Applied Therapeutics: The Clinical Use of Drugs..

[21] MacDonald JJ, Moore J, Davey V, Pickering S, Dunne T (2015). The weight debate.. J Intensive Care Soc..

[22] Chisholm MA, Vollenweider LJ, Mulloy LL, Jagadeesan M, Wade WE, DiPiro JT (2000). Direct patient care services provided by a pharmacist on a multidisciplinary renal transplant team.. Am J Health Syst Pharm..

[23] Chisholm MA, Mulloy LL, Jagadeesan M, Martin BC, DiPiro JT (2002). Effect of clinical pharmacy services on the blood pressure of African-American renal transplant patients.. Ethn Dis..

[24] Chisholm MA, Mulloy LL, Jagadeesan M, DiPiro JT (2001). Impact of clinical pharmacy services on renal transplant patients' compliance with immunosuppressive medications.. Clin Transplant..

[25] Di Cocco P, Okoye O, Almario J, Benedetti E, Tzvetanov IG, Spaggiari M (2020). Obesity in kidney transplantation.. Transpl Int..

[26] Singh D, Lawen J, Alkhudair W (2005). Does pretransplant obesity affect the outcome in kidney transplant recipients?. Transplant Proc..

[27] Hortal L, Fernandez A, Losada A, Lorenzo M, Baamonde E, Plaza C (2001). Study of the cyclosporine concentration at 2 hours in stable renal transplant patients and relation to body mass index.. Transplant Proc..

[28] Kasap B, Soylu A, Turkmen M, Kavukcu S, Bora S, Gulay H (2006). Effect of obesity and overweight on cyclosporine blood levels and renal functions in renal adolescent recipients.. Transplant Proc..

[29] Flechner SM, Kolbeinsson ME, Tam J, Lum B (1989). The impact of body weight on cyclosporine pharmacokinetics in renal transplant recipients.. Transplantation..

[30] Han SS, Kim DH, Lee SM, Han NY, Oh JM, Ha J (2012). Pharmacokinetics of tacrolimus according to body composition in recipients of kidney transplants.. Kidney Res Clin Pract..

[31] Shibata N, Hayakawa T, Hoshino N, Minouchi T, Yamaji A (1997). A predictive model for area under the concentration versus time curve of cyclosporin A using several routine monitoring results in renal transplant patients.. Biol Pharm Bull..

[32] Irtan S, Saint-Marcoux F, Rousseau A, Zhang D, Leroy V, Marquet P (2007). Population pharmacokinetics and bayesian estimator of cyclosporine in pediatric renal transplant patients.. Ther Drug Monit..

[33] Modlin CS, Flechner SM, Goormastic M, Goldfarb DA, Papajcik D, Mastroianni B (1997). Should obese patients lose weight before receiving a kidney transplant?. Transplantation..

[34] Jindal RM, Zawada ET (2004). Obesity and kidney transplantation.. Am J Kidney Dis..

[35] Dashti-Khavidaki S, Ghaffari S, Gohari M, Khatami MR, Zahiri Z (2016). Tacrolimus Dose Requirement in Iranian Kidney Transplant Recipients within the First Three Weeks after Transplantation.. Int J Organ Transplant Med..

